# 2023 European Thyroid Association Clinical Practice Guidelines for thyroid nodule management

**DOI:** 10.1530/ETJ-23-0067

**Published:** 2023-08-14

**Authors:** Cosimo Durante, Laszlo Hegedüs, Agnieszka Czarniecka, Ralf Paschke, Gilles Russ, Fernando Schmitt, Paula Soares, Tamas Solymosi, Enrico Papini

**Affiliations:** 1 Department of Translational and Precision Medicine, Sapienza University of Rome, Rome, Italy; 2 Department of Endocrinology, Odense University Hospital, Odense, Denmark; 3 M. Sklodowska-Curie National Research, Institute of Oncology Gliwice Branch, Gliwice, Poland; 4 Cumming School of Medicine, University of Calgary, Calgary, Alberta, Canada; 5 Thyroid and Endocrine Tumors Department, Pitié-Salpêtrière Hospital, Sorbonne University GRC N°16, Paris, France; 6 Faculty of Medicine of University of Porto, CINTESIS@RISE and Institute of Molecular Pathology and Immunology, University of Porto (Ipatimup), Porto, Portugal; 7 Institute of Investigation and Innovation in Health (I3S), Faculty of Medicine of the University of Porto, Porto, Portugal; 8 Endocrinology and Metabolism Clinic, Bugat Hospital, Gyöngyös, Hungary; 9 Department of Endocrine and Metabolic Diseases, Regina Apostolorum Hospital, Albano, Rome, Italy

**Keywords:** thyroid nodule, follow-up, ultrasound, fine-needle aspiration, surgery, minimally invasive treatment, molecular biology, treatment, management

## Abstract

With the widespread use of sensitive imaging techniques, which include neck visualization, a conspicuous number of thyroid nodules emerge and demand attention. Most lesions are benign, asymptomatic, and do not warrant treatment. In the case of cancer diagnosis, most are small, intrathyroidal and indolent neoplasms that can safely be managed conservatively. There is a pronounced need for more cost-effective, risk-adapted approaches to the management of this highly prevalent condition, taking the wishes of the patient into consideration. Thus, the present guidelines aim at providing a clinical practice guide for the initial workup and the subsequent management of adult individuals harboring thyroid nodules. Importantly, these guidelines are not intended to cover the management of thyroid malignancy. The manuscript and the specific recommendations were developed by reconciling the best available research evidence with the knowledge and clinical experience of the panelists and updating aspects of a number of previous European Thyroid Association guidelines.

## Introduction

A thyroid nodule is a discrete lesion within the thyroid gland that is ultrasonographically distinct from the surrounding thyroid parenchyma. With the widespread use of sensitive imaging techniques, which include neck visualization, a conspicuous number of thyroid nodules emerge and demand attention. Up to 60% of adults in the general population harbor one or more thyroid nodules (1). The likelihood of malignancy is an overriding concern, but the actual prevalence of cancer in unselected thyroid nodule populations generally ranges from 1 to 5%, with variation related to selection criteria and the population under evaluation, for example, whether papillary micro-carcinomas are included or not (2). Thus, most lesions are benign, asymptomatic, and do not warrant treatment. In the case of cancer diagnosis, most are small, intrathyroidal and indolent neoplasms (up to 53.6%, as shown in one contemporary large-scale study, in an unselected population) (3) that can safely be managed conservatively (4). Accepting that not all reasons for surgery are disclosed in the available literature, in European countries a proportion of such lesions are superfluously referred for surgery, leading to an unfavorable risk and cost–benefit ratio (5, 6, 7). A more conservative approach results in reduced cost as well as a lower risk of complications (8, 9). Implementation of active surveillance and minimally invasive techniques (MITs) has been limited and measured (10).

Overall, these observations cause concern for unwarranted expense and excess morbidity associated with thyroid nodule over-diagnosis and -treatment. This is exemplified by the abundantly demonstrated overdiagnosis of benign and malignant thyroid lesions without this leading to a significant lowering of thyroid cancer mortality (11, 12, 13). There is a pronounced need for more cost-effective, risk-adapted approaches to the management of this highly prevalent condition, taking the wishes of the patient into consideration. Based on the aforementioned, ultrasound (US) screening of asymptomatic adults is discouraged. Thus, the present guidelines aim at providing a clinical practice guide for the initial workup and the subsequent management of adult individuals harboring thyroid nodules. In the present manuscript, nodular thyroid disease includes both the solitary nodule, whether functioning or non-functioning, and multinodular goiter, independent of the patient having clinical goiter or not. Importantly, these guidelines are not intended to cover the management of thyroid malignancy. Notably, all recommendations should take into consideration the clinical setting, medical expertise, available technology, and patient preference. As a consequence, nationally adapted guidelines may well, in certain areas, deviate from this European Thyroid Association (ETA) guideline. Over the past years, the ETA has produced guidelines on the topics of US risk stratifications of thyroid nodules (14) and lymph nodes (15), molecular cytology diagnostics (16), as well as MIT for benign (17) and malignant nodules (18). The present document incorporates and updates aspects of these guidelines, where appropriate. For more in-depth information the reader is referred to the guidelines in question.

**Figure 1:**
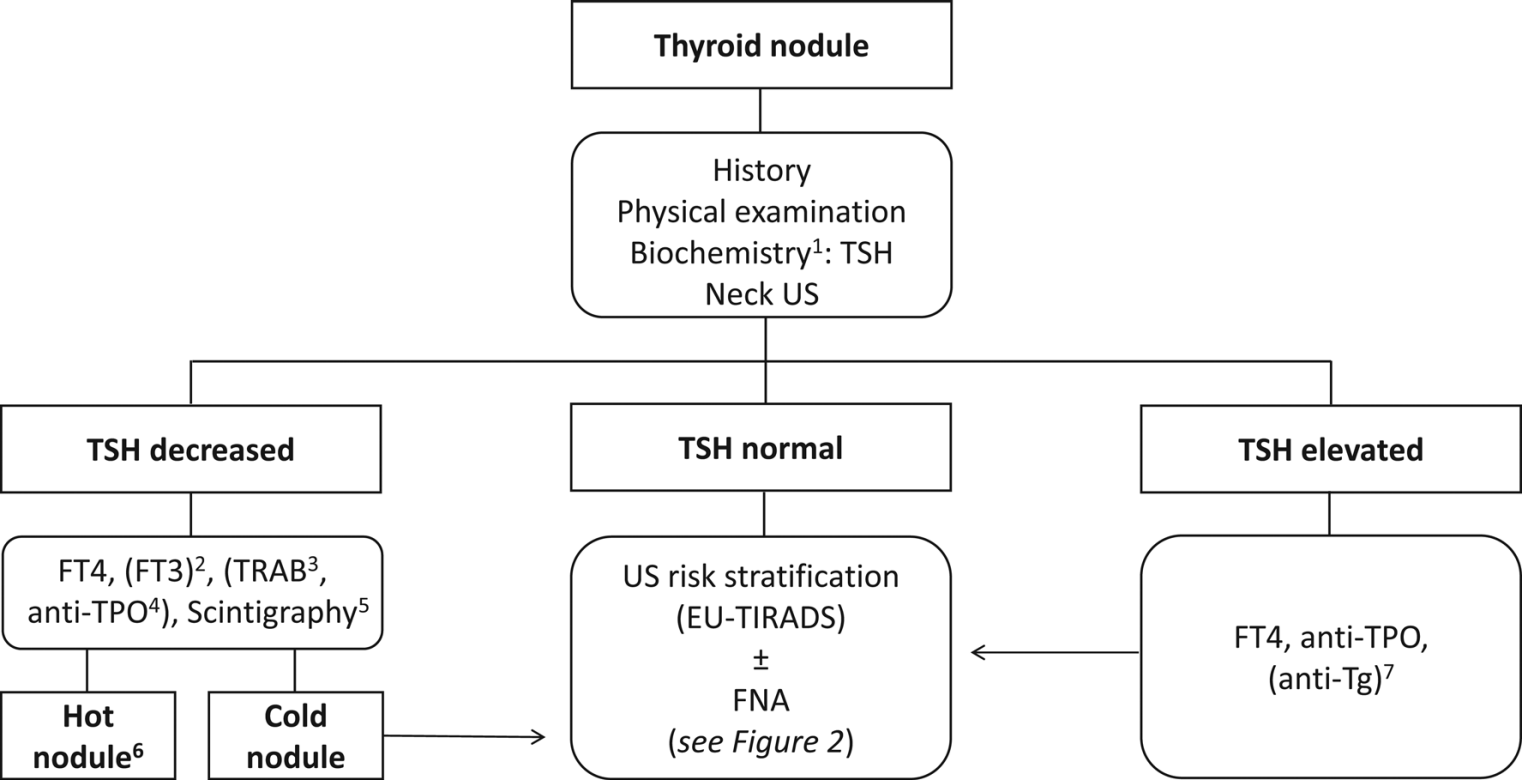
Initial evaluation for the investigation and diagnosis of the etiology of nodular thyroid disease. 1) Based on current evidence, the guideline panel cannot recommend for or against the routine use of calcitonin determination in the initial evaluation of a patient with thyroid nodule disease. Calcitonin determination should be considered in selected conditions (for details see the guideline text). 2) If the FT4 is normal, FT3 should be measured. 3) Based on the clinical context TSH receptor antibody determination may be considered to define the etiology of hyperthyroidism. 4) Consider TPOAb determination in case of clinical and US suspicion of thyrotoxicosis related to thyroiditis. 5) In current or previous iodine-deficient areas, the use of scintigraphy may be considered for nodular goiter and also for individuals with normal TSH. 6) See main text for management (paragraph ‘Radioiodine therapy’). 7) In case of clinical or US suspicion of chronic lymphocytic thyroiditis and negative TPOAb, Tg antibody determination may be considered. EU-TIRADS, European Thyroid Imaging and Reporting Data System; FNA, fine-needle aspiration; FT3, free tri-iodothyronine; FT4, free thyroxine; Tg, thyroglobulin; TPO, thyroid peroxidase; TRAB, TSH receptor antibody; TSH, thyroid-stimulating hormone; US, ultrasound.

**Figure 2:**
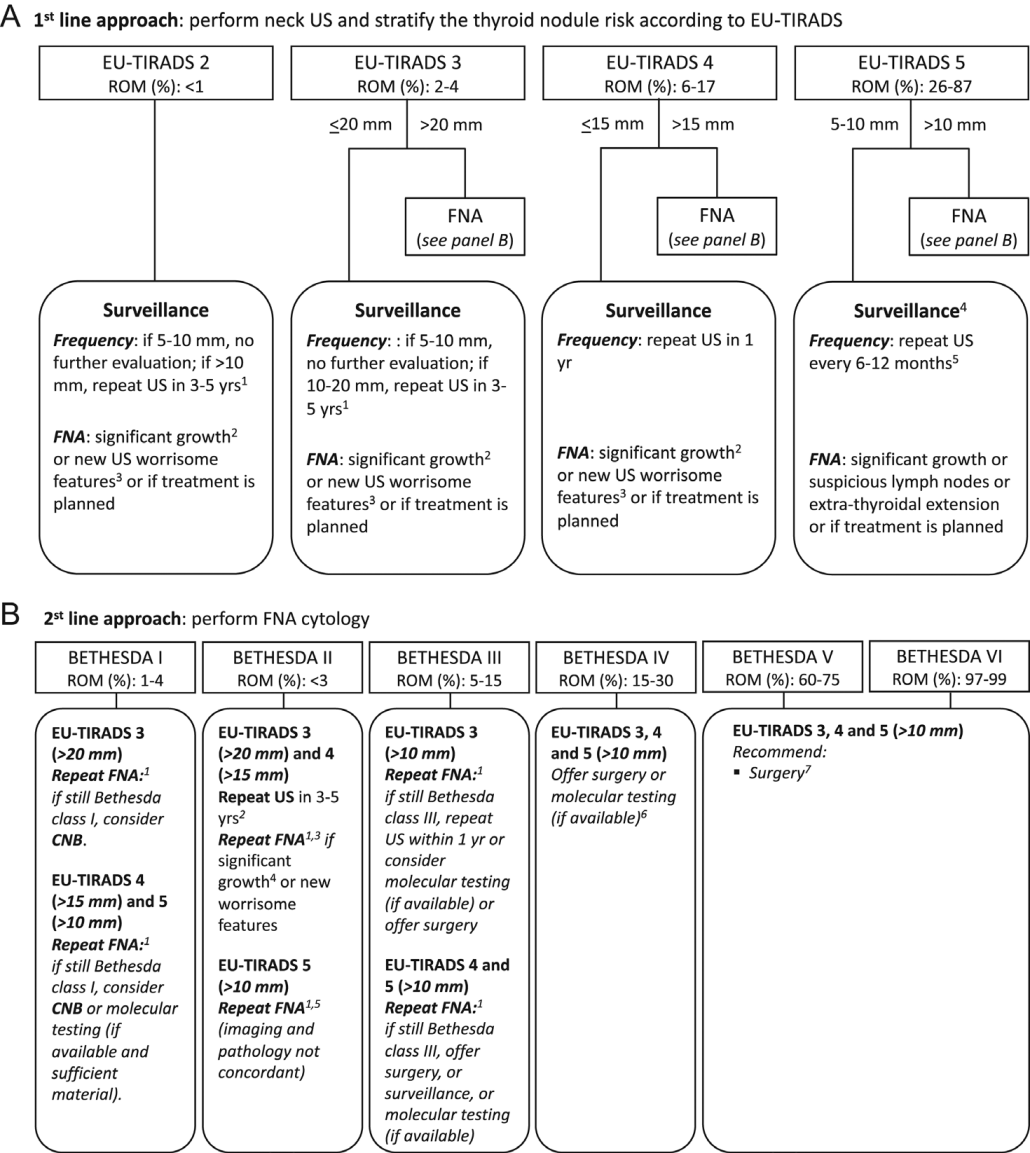
Diagnostic workup and the recommendations faced with a newly diagnosed thyroid nodule. Panel (A) describes the first-line approach, where clinical action is based on the US risk stratification. 1) There is no evidence for the length and modalities of follow-up and no consensus among the authors. Based on the ability to predict growth rate (mean change in the largest diameter over 5 years: 4.9 mm (95% CI, 4.2–5.5 mm)) (83), changes in TIRADS category which qualify for further workup (rate of change over 5 years: 6.3–8.3%) (126), risk of overlooking malignancy over the next 5 years (rate: 0.6%) (106), we find it appropriate to offer re-evaluation in 3–5 years. Independent of the Bethesda class, symptomatic nodules not offered treatment, for whatever reason, should be re-evaluated within 1 year. 2) An increase ≥20% in at least two nodule diameters with a minimum increase of 2 mm, or nodule volume increase >50% (35), or in case of local compression symptoms. 3) Irregular shape, irregular margins, microcalcifications, marked hypoechogenicity. 4) For 5–10 mm EU-TIRADS 5 nodules, FNA is recommended if there are suspicious lymph nodes, risk of extra-thyroidal extension, or location in worrisome areas (e.g. close to trachea, laryngeal nerve area). 5) If no changes are observed after 2 years, decreasing the intensity of follow-up may be considered. Panel (B) describes second-line approach, where the clinical action is based on the cytological report and US risk stratification. 1) When re-biopsy is considered relevant, the adequacy of the sample seems independent of the time interval between procedures. 2) There is no evidence for the length of and modalities with which to follow-up and no consensus among the authors. Based on the ability to predict growth rate (mean change in the largest diameter over 5 years: 4.9 mm (95% CI, 4.2–5.5 mm)) (106), changes in TIRADS category which qualify for further workup (rate of change over 5 years: 6.3–8.3%) (126), risk of overlooking malignancy over the next 5 years (rate: 0.6%) (106), we find it appropriate to offer re-evaluation in 3-5 years, with the potential of classifying/reclassifying and thereby allow stopping further follow-up. 3) No need to repeat the biopsy after two Bethesda class II results. 4) An increase ≥20% in at least two nodule diameters with a minimum increase of 2 mm, or nodule volume increase >50%, or in case of local compression symptoms. 5) A repeat benign cytology strongly reduces the likelihood of malignancy and favors surveillance. 6) In the case of 5–10 mm EU-TIRADS 5 nodules undergoing FNA and coming out as Bethesda class IV, surveillance may be offered as an alternative option. Consider close clinical follow-up in nodules smaller than 15 mm, with favorable ultrasound features. 7) In the case of 5–10 mm EU-TIRADS 3, 4, and 5 nodules undergoing FNA and classified as Bethesda class V or VI, surveillance or minimally invasive treatment may be offered as alternative options in the absence of suspected lymph node involvement or extra-thyroidal extension. CNB, core-needle biopsy; FNA, fine-needle aspiration; ROM, risk of malignancy; US, ultrasound.

Recommendations for thyroid nodule management are summarized in Table 1 and Figs. 1 and 2.

**Table 1:** Summary of recommendations.^a^

**Initial evaluation** Initial evaluation should include personal and family history, physical evaluation, thyroid function testing, and neck US assessment (*Ungraded good practice statement. Agreement: 9/9 (100%); round: 1*) Consider the use of a disease-specific patient-reported outcome (PRO) measure for evaluation of symptomatology (*Strength of recommendation: 1; quality of evidence: ØØØO. Agreement: 8/9 (88.9%); round: 1*)
**Thyroid ultrasound** Neck US, including the thyroid gland and the central and lateral cervical compartments, should be performed in all patients suspected of nodular thyroid disease (*Strength of recommendation: 1; quality of evidence: ØØOO. Agreement: 9/9 (100%); round: 1*) Describe nodule size, location, US features, and expected risk of malignancy using EU-TIRADS (*Strength of recommendation: 1; quality of evidence: ØØOO. Agreement: 9/9 (100%); round: 1*) In case of multinodularity, describe the details of all nodules with suspicious features (*Strength of recommendation: 1; quality of evidence: ØØOO. Agreement: 9/9 (100%); round: 1*) Doppler imaging, elasto-sonography, and CEUS may be considered as ancillary techniques (*Strength of recommendation: 2; quality of evidence: ØOOO. Agreement: 9/9 (100%); round: 1*) CEUS may be considered^b^ for defining the size and boundaries of the ablated area after minimally invasive procedures (*Strength of recommendation: 2; quality of evidence: ØØOO. Agreement: 8/9 (88.9%); round: 1*)
**Thyroid biopsy** Combine clinical factors, laboratory evaluation, and US risk stratification when defining the indication for FNA, in a shared decision with the patient (*Ungraded good practice statement. Agreement: 9/9 (100%); round: 1*) US guidance and use of either capillary action or suction is recommended when performing thyroid nodule FNA (*Strength of recommendation: 1; quality of evidence: ØØØO. Agreement: 9/9 (100%); round: 1*) FNA indication should be based on the following size cut-offs: EU-TIRADS 5: >10 mm EU-TIRADS 4: >15 mm EU-TIRADS 3: >20 mm (*Strength of recommendation: 2; quality of evidence: ØOOO. Agreement: 9/9 (100%); round: 1*) Repeat FNA should be considered in case of a first non-diagnostic sample (excluding the solitary cyst), a Bethesda class III cytology, discrepancy between US risk score (i.e. high risk) and cytological findings (i.e. benign cytology), and significant nodule growth^c^ (*Strength of recommendation: 1; quality of evidence: ØØØO. Agreement: 8/9 (88.9%); round: 1*) FNA is recommended in suspicious lymph nodes, with thyroglobulin or calcitonin washout dependent on phenotype (*Strength of recommendation: 1; quality of evidence: ØØØO. Agreement: 9/9 (100%); round: 1*) Core-needle biopsy should not be used as a first-line tool to assess thyroid nodules after US but could be considered a second-line procedure for specific conditions (*Strength of recommendation: 1; quality of evidence: ØØOO. Agreement: 8/9 (88.9%); round: 1*)
**Management of asymptomatic nodules not undergoing FNA** EU-TIRADS 2: 5–10 mm: no further evaluations >10 mm: re-evaluate the nodule in 3–5 years^d^ (*Strength of recommendation: 1; quality of evidence: ØØOO. Agreement: 9/9 (100%); round: 1*) EU-TIRADS 3 (<20 mm): 5–10 mm: no further evaluations 10–20 mm: re-evaluate the nodule in 3–5 years^d^ (*Strength of recommendation: 2; quality of evidence: ØOOO. Agreement: 9/9 (100%); round: 1*) EU-TIRADS 4 (<15 mm): Re-evaluate the nodule in 1 year (*Strength of recommendation: 2; quality of evidence: ØØOO. Agreement: 8/9 (88.9%); round: 1*) EU-TIRADS 5 (<10 mm):^e^ Re-evaluate the nodule every 6–12 months (*Strength of recommendation: 2; quality of evidence: ØØØO. Agreement: 8/9 (88.9%); round: 1*)
**Cytopathology-based management**
Correlate the cytological diagnosis with clinical, ultrasound and laboratory results (*Ungraded good practice statement. Agreement: 9/9 (100%); round: 1*)
Bethesda I EU-TIRADS 3 (>20 mm): repeat FNA. If still non-diagnostic, consider CNB. If still non-diagnostic, re-evaluate the nodule within 1 year or offer surgery EU-TIRADS 4 (>15 mm) and 5 (>10 mm): repeat FNA. If still non-diagnostic, consider CNB or, molecular testing (if available and sufficient material). If still non-diagnostic, offer active surveillance or surgery
(*Strength of recommendation: 2; quality of evidence: ØOOO. Agreement: 7/9 (77.8%); round: 2*)
Bethesda II EU-TIRADS 3 (>20 mm) and 4 (>15 mm): re-evaluate the nodule in 3–5 years^d^ EU-TIRADS 5 (>10 mm): repeat FNA^f^ (*Strength of recommendation: 2; quality of evidence: ØOOO. Agreement: 9/9 (100%); round: 2*) Bethesda III Repeat FNA regardless of EU-TIRADS class EU-TIRADS 3 (>10 mm), with repeat Bethesda III: re-evaluate the nodule within 1 year, consider molecular testing if available or offer surgery EU-TIRADS 4 and 5 (>10 mm), with repeat Bethesda III: offer surgery or active surveillance; molecular testing if available (*Strength of recommendation: 2; quality of evidence: ØOOO. Agreement: 9/9 (100%); round: 2*) Bethesda IV All nodules, regardless of EU-TIRADS class: offer surgery^g^; molecular testing if available (*Strength of recommendation: 2; quality of evidence: ØØOO. Agreement: 8/9 (88.9%); round: 1*) Bethesda V and VI All nodules, regardless of EU-TIRADS class: recommend surgery^h^ Active surveillance and MIT may be considered in patients with 5-10 mm nodules, in the absence of suspicious lymph nodes or risk of extra-thyroidal extension^i^ Multidisciplinary workup is warranted in case of advanced cancer (*Strength of recommendation: 1; quality of evidence: ØØØO. Agreement: 8/9 (88.9%); round: 1*)
**Molecular diagnostics of indeterminate thyroid nodule cytology** Molecular testing may be considered in cytologically indeterminate nodules, if available (*Strength of recommendation: 1; quality of evidence: ØØØO. Agreement: 9/9 (100%); round: 1*)
**Non-ultrasound imaging modalities** Thyroid scintigraphy should be performed when serum TSH is subnormal in order to diagnose functioning nodules and/or multinodularity, avoid FNA and determine eligibility for RAI as an alternative to surgery (*Strength of recommendation: 1; quality of evidence: ØØØO. Agreement: 9/9 (100%); round: 1*) The use of cross-sectional imaging (i.e. CT and MRI) in the study of thyroid nodules should be limited to the assessment of local extension or retrosternal growth of nodular goiter (*Strength of recommendation: 1; quality of evidence: ØØOO. Agreement: 9/9 (100%); round: 1*)
**Therapeutic options** ***Non-surgical approaches*** Clinical surveillance of benign thyroid nodules which do not require therapeutic intervention, should be followed up according to the management schedule illustrated in Table 1, section ‘*Management of asymptomatic nodules not undergoing FNA*,’ and Fig. 1 (*See related sections for grading and agreement*) Thyroid hormone treatment is not indicated in euthyroid individuals with nodular thyroid disease (*Strength of recommendation: 1; quality of evidence: ØØØO. Agreement: 9/9 (100%); round: 1*) Iodine and/or selenium supplementation is not indicated unless individuals are deficient in these micronutrients (*Strength of recommendation: 1; quality of evidence: ØØOO. Agreement: 9/9 (100%); round: 1*) RAI is recommended as an alternative to surgery and MIT in hyper-functioning solitary thyroid nodules (*Strength of recommendation: 1; quality of evidence: ØØØO. Agreement: 9/9 (100%); round: 1*) Consider RAI as an alternative to surgery in benign normo-functioning multinodular goiter (*Strength of recommendation: 2; quality of evidence: ØØOO. Agreement: 9/9 (100%); round: 1*) Consider EA as the first-line treatment for pure, or dominantly cystic, thyroid lesions (*Strength of recommendation: 1; quality of evidence: ØØØO. Agreement: 9/9 (100%); round: 1*) Consider TA for the treatment of solid benign thyroid nodules that cause local symptoms as an alternative to surgery and for cystic lesions that relapse after EA^l^ (*Strength of recommendation: 1; quality of evidence: ØØOO. Agreement: 8/9 (88.9%); round: 1*) Pre-MIT obtains a repeat benign cytological diagnosis, except for EU-TIRADS class 2 nodules, check vocal cord function, and consider bleeding disorders (*Strength of recommendation: 1; quality of evidence: ØØOO. Agreement: 8/9 (88.9%); round: 1*) After MIT, follow-up patients with clinical, biochemical and US assessments after 6 and 12 months. Re-evaluate the patient after 3–5 years (*Strength of recommendation: 1; quality of evidence: ØØOO. Agreement: 9/9 (100%); round: 1*)
***Surgical approach*** Surgery may be adopted in the following scenarios: Symptomatic nodular thyroid disease Nodules that have been classified as benign at cytology and/or US and become symptomatic over time Calcitonin levels higher than the established cut-offs (30) Responsive calcitonin after stimulation test in RET-mutated gene carriers Nodules with indeterminate cytology (Bethesda class III and IV) that are not suitable for active surveillance Nodules with a Bethesda class V and VI cytology (*Strength of recommendation: 1; quality of evidence: ØØØO. Agreement: 8/9 (88.9%); round: 1*)

^a^Each recommendation should take into consideration available expertise and technology, legislation, patient related (e.g. life-expectancy, comorbidity, preference, PRO) and nodule related (e.g. size and local symptoms) factors. The following information is shown in parentheses: the strength of recommendation, the quality of evidence, the number and percent of individuals who agreed with each recommendation, and the round in which the voters agreed on the definitions.

^b^Its use is limited because contrast agents are expensive, invasive, and not universally licensed for this purpose.

^c^An increase ≥20% in at least two nodule diameters with a minimum increment of 2 mm, or nodule volume increase >50%, or in case of local compression symptoms. This definition of growth reduces observer variation (35) and focuses the attention on management, whether benign or malignant.

^d^There is no robust evidence for the length of and modalities with which to follow-up and no consensus among the authors. Based on the ability to predict growth rate (mean change in the largest diameter over 5 years: 4.9 mm (95% CI, 4.2–5.5 mm)) (106), changes in TIRADS category which qualify for further workup (rate of change over 5 years: 6.3–8.3%) (126), risk of overlooking malignancy over the next 5 years (rate: 0.6%) (106), we find it appropriate to offer re-evaluation in 3–5 years, with the potential of classifying/reclassifying and thereby allow stopping further follow-up.

^e^If no changes are observed after 2 years, one may consider decreasing the intensity of follow-up.

^f^A repeat benign cytology strongly reduces the likelihood of malignancy and favors surveillance.

^g^In case of 5–10 mm EU-TIRADS 5 nodules undergoing FNA and coming out as Bethesda class IV, surveillance may be offered as an alternative option. Consider close clinical follow-up in nodules smaller than 15 mm, with favorable ultrasound features.

^h^Based on the Bethesda system for reporting thyroid cytopathology, lymphoma will mainly be reported as Bethesda V and surgery may not be recommended.

^i^This scenario occurs when a patient has an FNA, despite not being recommended, following a shared decision process between patient and physician.

^l^In multinodular nodular thyroid disease, TA should be restricted to patients with a well-defined dominant nodule or to those who are not suitable for thyroid surgery or RAI, as a palliative option.

CEUS, contrast-enhanced ultrasound; CNB, core-needle biopsy; CT, computed tomography; EA, ethanol ablation; FNA, fine-needle aspiration; MIT, minimally invasive techniques; MRI, magnetic resonance imaging; PRO, patient-reported outcome; RAI, radioactive iodine; TA, thermal ablation; TIRADS, Thyroid Imaging and Reporting Data System; US, ultrasound.

## Methodology and grading of evidence

The Executive Committee of the ETA, upon consultation with its Guideline Board, commissioned the development of this guideline to a multidisciplinary team led by two chairpersons (C D, L H). The task force consisted of four endocrinologists (C D, L H, R P, E P), one internist/clinical cytologist (T S), one endocrine surgeon (A C), a radiologist (G R), one pathologist (F S), and a biologist (P S). All panelists had to be members of the ETA and be experts in nodular thyroid disease. In hindsight, the document could have benefitted from including even more specialists, for example, a nuclear medicine physician. However, we have attained this by incorporating the comments from nuclear medicine experts within the ETA.

The literature considered was retrieved based on a systematic search on the MEDLINE database, through the PubMed search engine. The manuscript and the specific recommendations were developed by reconciling the best available research evidence with the knowledge and clinical experience of the panelists. The grading of recommendations, assessment, development, and evaluation (GRADE) framework was used for grading the quality of evidence and making clinical practice recommendations (19). The quality of a body of evidence was rated as high (ØØØØ), moderate (ØØØO), low (ØØOO), or very low (ØOOO), while the strength of a recommendation was categorized as either strong (recommended for all or almost all patients; indicated by 1) or weak (different choices may be appropriate for some patients and settings; indicated by 2). The statements that panelists considered worthy of being presented, but inappropriate for rating the evidence despite a considerable and compelling amount of indirect evidence, are described as ‘ungraded good practice statement’ (20). Consensus on all the recommendations was reached following a modified Delphi process, which involved two rounds of voting using an online survey tool (Google Forms platform). Panelists rated each recommendation on a 5-point scale: strongly agree, agree, neutral, disagree, or strongly disagree. Consensus was defined as >80% of voters (that is a minimum of eight out of nine voters) strongly agreeing, agreeing or being neutral with a statement. The total number and percent of individuals who agreed with each recommendation and the round in which agreement was achieved are reported in Table 1. The final draft of the manuscript was sent to the Guideline Board for comments and thereafter posted on the ETA website for four weeks for critical evaluation by the ETA members. All proposed changes and comments were taken into consideration within the guideline task force, and the resulting changes were incorporated into the final document submitted to the *European Thyroid Journal*, after approval by the Guideline Board.

## Initial evaluation

The initial evaluation of any patient suspected of nodular thyroid disease includes the combination of personal and family history, physical examination, evaluation of thyroid function, and US of the neck. This should take into consideration pre-treatment symptomatology, ideally including a thyroid specific patient-reported outcome (PRO) (21), which has been cross-culturally validated (22) and for which instrument minimally important change in the quality of life has been determined (23), allowing the use for treatment effect evaluation.

As a minimum, laboratory assessment needs to include thyroid-stimulating hormone (TSH) measurement. If TSH is decreased, we recommend determining free thyroxine (FT4). If the latter is normal, free tri-iodothyronine should be measured. Based on the clinical context, TSH receptor antibody determination may be considered to define the etiology of hyperthyroidism. If TSH is elevated, FT4 and anti-thyroid peroxidase (TPO) antibodies should be measured to aid in the classification of the etiology of thyroid dysfunction. In case of clinical or US suspicion of chronic lymphocytic thyroiditis and negative anti-TPO antibodies, measurement of anti-thyroglobulin (Tg) antibodies may be considered (Fig. 1) (24).

Using calcitonin for medullary thyroid carcinoma (MTC) screening in unselected thyroid nodule populations provides an early diagnosis and thereby potentially improves prognosis (25, 26). However, the value of such screening is still under debate, based on the fact that at most 1 in 200 screened individuals will have MTC (27) and that some might also have been diagnosed without routine screening. In the following scenarios, calcitonin evaluation is appropriate: patients with thyroid nodules scheduled for surgery or MIT (17, 28); thyroid nodules with indeterminate cytology or suspicious US findings (29). Calcitonin determination should be performed in case of personal or family history of medullary thyroid cancer or multiple endocrine neoplasia type 2 (27). Cut-off points to separate non-medullary reasons for increased calcitonin from medullary thyroid carcinoma have been established (>30 pg/mL in females and >34 pg/mL in males, even if several variables may affect this threshold) (30).

Serum Tg and Tg antibody determination has no role in the initial evaluation of nodular thyroid disease (31).

## Thyroid ultrasound

US is more sensitive and specific than palpation for the evaluation of nodular thyroid disease (32).

Diagnostic thyroid and neck US should be performed in all patients clinically suspected of having nodular thyroid disease, or if a nodule is incidentally detected using another imaging modality (e.g. US scan of carotid arteries, computed tomography (CT) of the neck) (33).

## Anatomical regions to be evaluated

**Figure 3:**
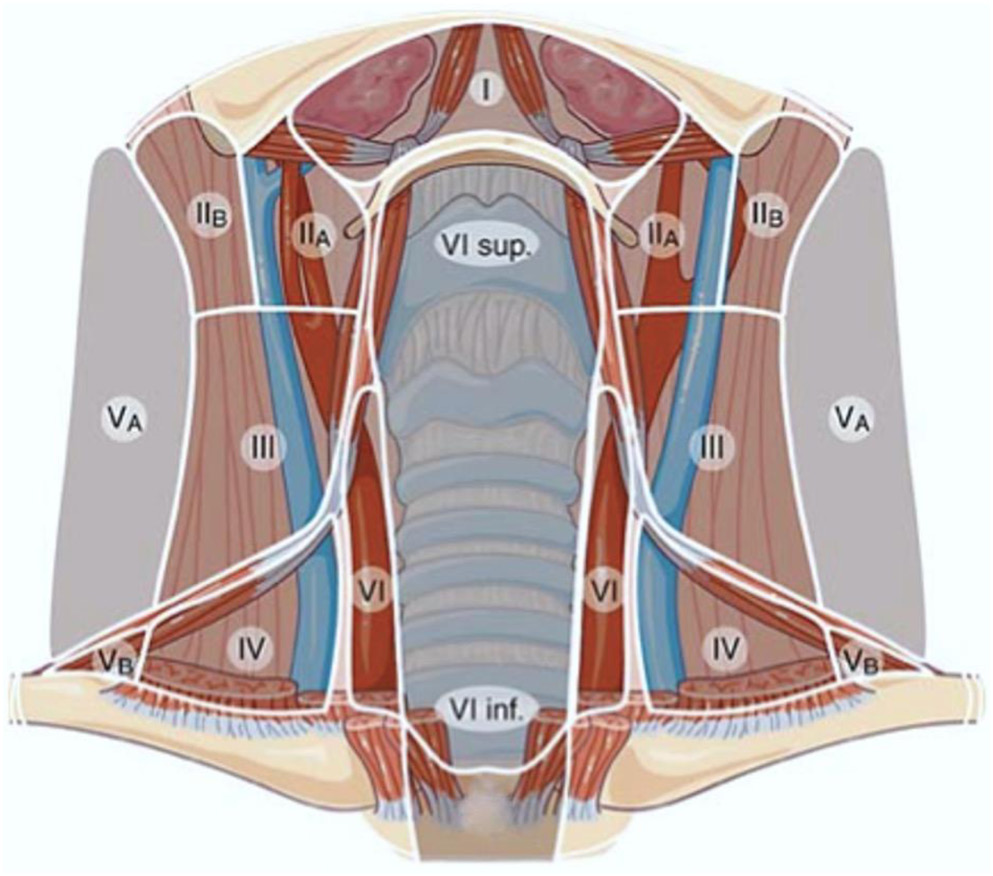
Diagram for making the location of lymph nodes using the levels nomenclature. Only a small portion of level VII can be visualized by US. For this reason, level VII was merged into level VI (modified from reference (15)).

- The thyroid bed and anterior neck from hyoid bone to sternal notch and below, if accessible.
- From levels II to V in the lateral neck and level VI in the central neck for lymph nodes (Fig. 3).

A high-frequency linear probe (e.g. up to 14 MHz) is adequate for exploring these regions. To visualize the inferior pole of an intrathoracic thyroid a convex probe (frequency range: 2.5–5.0 MHz) may be useful (34).

### Applying thyroid ultrasound in the evaluation of nodular thyroid disease

#### First examination

- Assess the presence, location, size, and features of nodule(s) and thyroid lobes. Details of what is expected in the US report are provided in Table 2. For a description of thyroid nodule features, the use of an appropriate lexicon is warranted (Appendix, see section on supplementary materials given at the end of this article).

**Table 2:** Elements of thyroid ultrasound reporting in nodular thyroid disease.

Thyroid lobes	Echogenicity
	Size (three diameters and volume)
	Presence of substernal extension or compression of cervical structures
Nodule	Size (three diameters and volume)
	Location (according to the three axes)
	Echogenicity
	Composition
	Suspicious and non-suspicious signs if present^a^
	Possible extrathyroidal extension
Which discrete lesions should be described?	Nodules larger than 10 mm.
	Nodules between 5 and 10 mm with suspicious signs
How many nodules should be described in detail?	The largest one and those with suspicious signs if the number of nodules is >3 in a lobe^b^
Pathological^c^ lymph nodes if present	Location, three diameters, features

^a^Suspicious ultrasound characteristics: microcalcifications, irregular margins, nonparallel orientation, marked hypoechogenicity of the solid part. Non-suspicious ultrasound characteristic: thin halo, macrocalcification (specify rim calcification)

^b^The propensity to offer surgery increases with number of suspicious nodules.

^c^Features of high suspicion are the presence of cystic areas, microcalcifications, thyroid tissue-like appearance, and anarchic vascularity in the absence of a visible hilum (15).
- Visualize any intrathoracic extension and its relationship with the cervical structures.
- Stratify the risk with the use of the European Thyroid Imaging and Reporting Data System (EU-TIRADS) (Table 3).

**Table 3:** EU-TIRADS categories with corresponding malignancy risks and indication of fine-needle aspiration cytology.

Category	Ultrasound features^a^	Estimated malignancy risk according to ETA guidelines (%)	Observed malignancy risk vs surgery (127)	FNA^b^
*EU-TIRADS 1*: normal	No nodule	None		No
*EU-TIRADS 2*: benign	Pure cyst Entirely spongiform	0	1.4	No, unless scheduled for treatment
*EU-TIRADS 3*: low risk	Iso/hyperechoic No feature of high suspicion	2–4	3.5	If >20 mm
*EU-TIRADS 4*: intermediate risk	Mildly hypoechoic No feature of high suspicion	6–17	17	If >15 mm
*EU-TIRADS 5*: high risk	At least one of the following features of high suspicion: Irregular shape Irregular margins Microcalcifications Marked hypoechogenicity	26–87	87.7	If >10 mm^c^

^a^If difficulties with ascertaining the presence of features of high suspicion, we suggest classifying these nodules as EU-TIRADS 4.

^b^FNA should be performed in nodules irrespectively of EU-TIRADS score if either pathological lymph nodes are present or the nodule is suspicious of extra-thyroidal extension.

^c^For 5–10 mm high suspicion nodules, FNA should be considered if there are suspicious lymph nodes or if there is suspicion of extra-thyroidal extension.

FNA, fine-needle aspiration; TIRADS, Thyroid Imaging and Reporting Data System.
- Investigate for the presence of suspicious lymph nodes.
- Select nodules to be addressed to fine-needle aspiration (FNA) biopsy.

#### Follow-up of untreated thyroid nodule(s)

- Monitor growth (an increase ≥20% in at least two nodule diameters with a minimum increase of 2 mm, or nodule volume increase >50% at the time of re-evaluation) of thyroid nodule(s) (35) (Fig. 2).
- Monitor US feature changes that may modify risk stratification.
- Monitor lateral neck lymph nodes.
- Re-evaluate in case of the appearance of local pressure symptoms and/or voice changes.

### Complementary ultrasound techniques

#### Doppler imaging

The usefulness of the Doppler vascular pattern for defining the risk of malignancy (ROM) of thyroid nodules is controversial (36, 37, 38). Even if more sensitive techniques are now available (e.g. super-resolution, microvascular imaging) (39), their role remains unclarified. However, besides providing complementary information, Doppler imaging may be indicated in order to differentiate between cases where vascularization is diminished or absent (e.g. thick colloid, cystic or necrotic nodules) from solid nodules (40). So, it may indirectly be useful for risk stratification and for guiding FNA and minimally invasive procedures in mixed thyroid lesions.

#### Elasto-sonography

The role of elastography remains unsettled. The studies concerning the usefulness of these techniques have not resolved a consensus on their use or reporting (41, 42, 43). Importantly, while the classical variant of papillary thyroid carcinoma (PTC) has demonstrated high stiffness, other variants of PTC and follicular thyroid carcinoma (FTC) may show a normal stiffness (44). Thus, the contribution of this method to the standard US imaging does not justify its routine use and inclusion in the risk-stratification systems (RSSs).

#### Contrast-enhanced ultrasound

A few meta-analyses report that contrast-enhanced ultrasound (CEUS) has a rather high positive and negative predictive value for the assessment of the ROM (45, 46). However, authors have reported inconsistency across studies and publication bias. Thus, even if these methods may add some information to gray-scale US examination, their use is limited because contrast agents are expensive, invasive, and not universally licensed for this purpose. However, CEUS provides a clear depiction of the ablated areas after thermal ablation (TA) of thyroid nodules and offers an advantage for guiding the need for repeat treatments (18).

### Thyroid Imaging and Reporting Data System

TIRADS scores have been proposed for improving inter-observer reproducibility (47, 48) in the description of US features and quantifying malignancy risk of thyroid nodules. Additional goals are to unify the reports, to facilitate their understanding and to standardize the management recommendations. Currently, there are several stratification systems, either ‘pattern-based’ – when all the features of the nodule are taken into account to analyse its risk – or ‘point-based,’ when points are assigned to each US sign (49). No system has consistently demonstrated superiority over another, and the inter-observer reproducibility of the scores is similar (50). Each system provides recommendations for FNA, based on the score and size of the nodules. As all TIRADS reduce the number of unnecessary FNAs, all relevant specialty societies endorse using TIRADS. In Europe, the preferred system is the EU-TIRADS (Table 2) (14, 51).

Even if TIRADS scores provide quantitative malignancy risk stratification, users should be aware of the following:

- TIRADS have been designed and mainly tested for PTCs, although they are proposed to estimate the malignancy risk of any thyroid neoplasm. The sensitivity for the detection of the classical variant is excellent but decreases substantially for the follicular variant and even more for FTCs (52, 53). Accuracy in identifying medullary thyroid carcinomas is debated (54).
- Misdiagnoses may occur, especially in cystic nodules and in sub-acute as well as chronic thyroiditis (55, 56).
- Composition of the nodule is not included in the cardinal features of the EU-TIRADS. However, the users should consider that the ROM is higher in completely solid than mainly cystic ones (57).
- Ultrasound features suggestive of extra-thyroidal extension (i.e. capsular bulging, disruption, or abutment by the thyroid nodule) are not included in the cardinal features of the EU-TIRADS. However, they should be described in the report as they are associated with a higher ROM and should prompt FNA irrespectively of EU-TIRADS score (14).
- When using EU-TIRADS and in case of difficulties with ascertaining the presence of features of high suspicion, we suggest classifying these nodules as EU-TIRADS 4 (14).
- TIRADS scores do not include lymph node evaluation. Cervical lymph nodes should be described according to the 2013 ETA guidelines terminology as normal, indeterminate, or suspicious, and located using the six cervical levels nomenclature (Fig. 3). Features of high suspicion are the presence of cystic areas, microcalcifications, thyroid tissue-like appearance, and anarchic vascularity in the absence of a visible hilum (15).

## Thyroid biopsy

### Fine-needle aspiration

Having obtained clinical, biochemical and US evaluation, and in a dialogue with the patient, a decision regarding the indication for FNA can be made. How to perform the FNA is beyond the scope of this guideline and we refer to a recent video (58). As a rule, FNA should be performed under US guidance. Each US RSS has its own cut-offs for guiding indications for FNA cytology, and there is a continuous debate on the optimum threshold (59). These recommendations have been shown to reduce the number of superfluous FNAs. For instance, compared prospectively in 477 patients, the performances of five internationally endorsed sonographic classification systems (those of the American Thyroid Association (ATA), American Association of Clinical Endocrinologists (AACE)-American College of Endocrinology (ACE)-Associazione Medici Endocrinologi (AME), American College of Radiology (ACR), ETA, and Korean Society of Thyroid Radiology), application of the FNA criteria would have reduced the number of biopsies performed by 17.1–53.4% (17.1% using K-TIRADS, 30.7% using EU-TIRADS, 34.9% applying AACE/ACE/AME, 43.8% for ATA, and 53.4% with ACR TIRADS). The percentage of missed carcinomas in nodules >1 cm was low comprising between 2.2% for ACR TIRADS and 4.1% for ATA TIRADS (60). Thus, all RSSs seem effective to reduce the number of unnecessary FNAs, at the expense of temporarily postponing the diagnosis of a minute proportion of carcinomas (60, 61, 62). The diagnosis of such carcinomas will be postponed until they eventually grow and are diagnosed after reaching the cut-off threshold defined for FNA, according to their US risk category. There is no evidence of this strategy implying a significant loss of quality of life or an increase in morbidity and mortality.

The indications for FNA, based on EU-TIRADS, and the factors that may influence this choice are described in Tables 3 and 4, respectively. Severe coagulation disorders represent a contraindication to FNA, while the use of anticoagulant therapy does not, as long as INR is below 3. Antiaggregant therapy is not an absolute contraindication to FNA.

**Table 4:** Criteria other than size and US risk level, which strengthen or weaken the indication for fine-needle aspiration.

	Strengthens FNA	Weakens FNA
Clinical factors	Male sex Young age Solitary nodule Compressive symptoms related to the nodule Family history of medullary thyroid cancer or MEN2 Head and neck radiation during childhood Planned thyroid or parathyroid surgery Patient preference	Long personal history of stable or slowly growing MNG Limited life expectancy Significant comorbidity Patient preference Family history of benign nodular thyroid disease
Genetic factors	Monogenic syndromic thyroid susceptibility Strong family history of thyroid cancer (>2 relatives)	
Biological tests	Elevated serum calcitonin Calcitonin responsive to stimulation test in RET gene carriers	Subnormal thyrotropin
Nuclear medicine imaging	18-FDG uptake MIBI uptake	Autonomous nodules on isotope scan

FDG, fluorodeoxyglucose; FNA, fine-needle aspiration; MEN2, multiple endocrine neoplasia type 2; MIBI, methoxy-isobutyl-isonitrile; MNG, multinodular goiter.

Unless highly suspicious for malignancy, hyperfunctioning thyroid nodules should not be biopsied. Scintigraphy should be performed in case of subnormal serum TSH, with or without elevated free thyroid hormones (24). In certain situations, scintigraphy may be warranted also when TSH is normal (e.g. in current or formerly iodine deficient regions and in case of a multinodular goiter). The reasons mainly being to decide eligibility for FNA and/or radioactive iodine (RAI) treatment.

FNA should be repeated in case of:

- a first non-diagnostic sample (63);
- a Bethesda class III cytology (64);
- discrepancy between US risk score (i.e. high risk) and cytological findings (i.e. benign cytology).

### Core-needle biopsy

Core-needle biopsy (CNB), performed with a large bore spring-activated device, may provide a micro-histological sample from thyroid lesions. There are no clear advantages of using CNB, a more invasive and expensive procedure compared to FNA, based on cost and risk–benefit analysis (65).

CNB may be considered in the following situations:

- repeat inadequate FNA as an alternative to diagnostic surgery (66);
- repeat Bethesda class III cytology (67);
- when histological assessment can improve pre-operative diagnosis (e.g. suspicion of poorly differentiated or undifferentiated thyroid cancer, thyroid lymphoma, thyroid metastases) (68).

The only available pathology reporting system has been proposed by the Korean thyroid CNB study group (69).

### Wash-out thyroglobulin, calcitonin, and parathyroid hormone determination

In patients suspected of lymph node metastases, diagnostic confirmation should be obtained by US-guided FNA, before offering therapy. In the case of differentiated thyroid cancer, Tg washout determination should be added, and in the case of medullary thyroid cancer, calcitonin measurement (70). FNA-Tg washout levels in metastatic thyroid lymph nodes are usually much higher than in the circulation and allow a definitive diagnosis (71). In some cases of small nodules with normal serum calcitonin but cytological suspicion of medullary thyroid cancer, or in case of elevated serum calcitonin, calcitonin washout assessment of the nodule can be useful (72). In the rare cases of intranodular parathyroid adenomas, parathyroid hormone determination in FNA washout may confirm the clinical suspicion (73).

## Pathology

### Cytopathology

Thyroid cytopathology should be reported using a widely accepted and endorsed classification system. Currently, the most widely used system is ‘The 2017 Bethesda System for Reporting Thyroid Cytopathology’ (TBSRTC), enjoying wide acceptance internationally (74). Advantages include well-defined ROM rates, management algorithms linked to each diagnostic category, and integration of molecular tests in the reporting (75). TBSRTC should be integrated into the local setting, with local calculation of ROM based on demography, including local thyroid cancer incidence. There are clearly formulated requirements for cytopathology quality control (76). The details are beyond the scope of this document. Table 5 shows the distribution of benign and malignant entities across the different Bethesda categories.

**Table 5:** Distribution of diagnoses across the Bethesda categories. The third edition of the Bethesda system has been released after the first online appearance of the current manuscript (131). It provides an updated summary of the reporting system and refined estimates of the risk of malignancy, which are therefore slightly different from those reported in Table 5.

Bethesda categories	Definition of Bethesda categories	Subclassification		Expected frequency (range)	Estimated malignancy risk (NIFTP not cancer)
		Benign entities	Malignant entities		
Bethesda I	Non-diagnostic	NA	NA	3–11%	5–10%
Bethesda II	Benign	Adenomatoid/hyperplastic/colloid nodule Lymphocytic thyroiditis Subacute granulomatous thyroiditis Acute thyroiditis Graves’ disease	PTC microcarcinomas in benign nodules	55–74%	0–3%
Bethesda III	Atypia of undetermined significance or follicular lesion of undetermined significance (AUS/FLUS)	Cyst lining cells Hashimoto’s thyroiditis with cellular atypia (both follicular and lymphocytic atypia) Adenomatoid nodule (cellular with microfollicular proliferation) Parathyroid adenoma (microfollicular structures) Hürthle cell hyperplasia with lack of colloid	PTC, especially follicular variant; well-differentiated follicular carcinoma; Hürthle cell carcinoma; lymphoma	5–15%	10–30%
Bethesda IV	Follicular neoplasm or suspicious for follicular neoplasm (FN/SFN)	Adenomatoid nodule (cellular with microfollicular proliferation) Parathyroid adenoma (microfollicular structures) Hürthle cell hyperplasia with lack of colloid Follicular-patterned cases with mild nuclear changes (increased nuclear size, nuclear contour irregularity, and/or chromatin clearing), and lacking true papillae and intranuclear pseudo-inclusions	PTC, especially follicular variant; well-differentiated follicular carcinoma; Hürthle cell carcinoma	2–25%	25–40%
Bethesda V	Suspicious of malignancy	Hashimoto’s thyroiditis with cellular atypia	Features suspicious for PTC, MTC, lymphoma, or other malignancy	1–6%	50–75%
Bethesda VI	Malignant	Hashimoto’s thyroiditis with cellular atypia	Features *conclusive* for malignancy: PTC (true papillae, psammoma bodies, nuclear pseudo-inclusions) MTC, poorly differentiated/ATC, non-endocrine malignancy (squamous cell, lymphoma, metastatic)	2–5%	97–99%

ATC, anaplastic thyroid carcinoma; MTC, medullary thyroid carcinoma; NA, not applicable; NIFTP, noninvasive follicular thyroid neoplasm with papillary-like nuclear features; PTC, papillary thyroid carcinoma.

#### Minimum requirements for a thyroid FNA cytopathology report

- Identification of the patient
- Imaging findings and, if available, TIRADS score
- Adequacy of the sample
- Microscopic description of the material including cellular and colloid components
- Ancillary testing (if performed)
- Reporting category and subclassification (specific diagnosis)
- The local ROM of the diagnostic category

### Immunocytochemistry

The morphology remains the cornerstone to distinguish benign from malignant nodules (77). HBME-1, Galectin-3, and CK19 immunostaining are more frequently positive in thyroid carcinomas, but their expressions are variable, and no single stain has sufficient sensitivity and specificity to be recommended for routine practice (78). There are recent studies applying antibodies to detect genetic alterations such as BRAF V600E (VE1), Pan-Trk, and ALK among others (79). However, they are not recommended for routine use. In some special situations, stains for calcitonin, Tg, and TTF1 can be useful, for example, in FNA of suspicious metastatic cervical lymph nodes (80).

## Molecular diagnostics applied to cytology

Using an integrated approach combining careful clinical, US, and cytology malignancy risk assessment with the local outcome and test performance data, molecular testing may improve diagnostic outcomes for thyroid nodules by identifying patients with indeterminate cytology as most likely benign (81). Based on local integrated diagnostic pathway outcome data, this strategy may obviate diagnostic surgery or identify patients with a high likelihood of malignancy, allowing surgical treatment (16).

Currently, available molecular FNA tests are based on examining for somatic mutations, evaluation of gene expression, and microRNA-based classifiers (82, 83, 84, 85, 86). For details of the ThyroSeq, the Afirma Genomic Sequencing Classifier (GSC) and the ThyGeNEXT/ThyraMIR, see Table 6. Up to 13.4% avoided diagnostic surgeries have been reported for the ThyroSeq and the Afirma GSC (87). ThyroidPrint is a gene expression classifier based on the interrogation of only ten mRNAs (83). ThyroSPEC™ is a MALDI-TOF mass spectrometry-based mutation detection panel that detects the most prevalent 117 point mutations and 23 gene fusions in thyroid cancer (88).

**Table 6:** Summary of genetic tests for aiding diagnosis of thyroid cancer in FNA cytology.

	Afirma GSC	ThyroSeq v3	ThyGeNEXT/ThyraMIR	ThyroidPrint
Type of test	RNA NGS (mRNA expression)	Targeted DNA and RNA NGS	Targeted NGS + miRNA expression	Quantitative real-time PCR (mRNA expression)
Biomarkers	1115 genes (expression) + mutation hotspots + fusions + LOH	112 genes + >120 fusions + 10 CNA + 19 genes (expression)	10 genes + 28 fusions + 10 miRNA (expression)	10 genes
NPV in marketing study (%)	96%	97%	95%	95%
PPV in marketing study (%)	47%	66%	74%	78%
Sensitivity in marketing study (%)	91%	94%	93%	91%
Specificity in marketing study (%)	68%	82%	90%	88%
Sample size Bethesda III, IV (n)	114, 76	154, 93	92, 86	117, 153
Advantages	Some independent validation studies	Most comprehensive mutation and CNA coverage, highest NPV in marketing study of commercially available tests	Best ROM stratification for *RAS-*positive nodules	Marketing study included a trial in South America and a trial in North America, highest PPV in marketing study of commercially available tests
Disadvantages	Mutation coverage is less sensitive because it uses RNA rather than DNA sequencing	A single-center study has shown a doubling in indeterminate thyroid nodule diagnosis following the implementation of ThyroSeq (128)	A ‘moderate’ test result in 21% of samples provides no clarity on diagnosis since the moderate category has a 39% risk of malignancy	No mutation data, no independent validation to date
Validation study	Patel *et al.* (2018) (84)	Steward *et al.* (2019) (85)	Lupo *et al.* (2020) (86)	Zafereo *et al.* (2020) (129)
Validation concerns	Post-marketing studies have conflicting results on NPV as resected nodules in the validation cohort are not representative of all indeterminate thyroid nodules (130). This results in unclear real-world benefit. In case of availability of similar post-marketing studies for the ThyroSeq or ThyGeNEXT/ThyraMIR or ThyroidPrint tests, a similar problem would likely also appear for these tests.	Few post-marketing studies result in unclear real-world benefit, since they have been concentrated at tertiary centers not representative of all practices.	No independent validation means there is no evidence of reproducibility of the diagnostic performance reported. Retrospective design of the validation study.	No independent validation means there is no evidence of reproducibility of the diagnostic performance reported. The ‘kit’ design rather than centralizing testing introduces the potential risk of variability when the test is performed in different labs.
Caveat	Arguments that unnecessary surgeries are avoided based on NPV/BCR incorrectly assume that all indeterminate thyroid nodules would undergo diagnostic surgery in the absence of molecular testing. If each positive molecular test result triggered surgery, implementation of molecular testing would substantially increase overtreatment. For *RAS* mutations, see text (’Molecular diagnostics applied to cytology’).			

CNA, copy number alteration; FNA, fine-needle aspiration; GCS, Genomic Sequencing Classifier; LOH, loss of heterozygosity; miRNA, microRNA; NGS, next-generation sequencing; NPV, negative predictive value; PCR, polymerase chain reaction; PPV, positive predictive value; ROM, risk of malignancy.

Although a fully comprehensive genomic profile of thyroid FNAs is offered by centralized laboratories in North America, recent efforts in other countries have concentrated on developing local laboratory-developed thyroid molecular tests in research settings of a few centers in Europe (89, 90, 91) and East Asia (92) and a publicly funded test in Canada (88) with promising results.

Notably, benign lesions frequently exhibit *RAS* mutations and have been reported positive for *PAX8/PPARG* rearrangements (93). The pronounced variation in reports of the prevalence of these and other biomarkers in benign lesions suggests the need for data with sufficient sample size and histologic evaluation according to WHO guidelines, and caution when including these markers especially in binary instead of complementary diagnostic decisions to avoid overtreatment of patients with benign thyroid tumors. Considerable inter-observer variation for differentiating benign follicular cell-derived tumors and minimally invasive FTCs hamper translational studies assessing the value of molecular markers that aim to improve presurgical diagnosis because the histological reference for these studies can be ambiguous or discrepant.

Currently, molecular FNA testing of indeterminate FNA outside of the USA is limited to mainly research use of local laboratory-developed tests and a publically funded test in Alberta/Canada. Molecular tests marketed in the USA are currently not used as reimbursed tests outside of the USA. Possible reasons for this are health care system differences, issues pertaining to lack of independent validation studies, lack of long-term outcome studies for ‘benign’ molecular tests, and the high cost that currently limits their use outside the USA.

Whether the comprehensive molecular profile of thyroid nodules can provide prognostic information and guide the extent of surgery is still debated. However, high-risk molecular profiles (e.g. coexistence of either BRAF p.V600E or RAS mutations with late-hit mutations like those in TERT promoter, PIK3CA or TP53 genes) have been strongly associated with the presence of distant metastases in DTC patients (94), thus increasing the odds of an indeterminate thyroid nodule with high-risk mutations being aggressive cancer.

## Non-ultrasound imaging modalities

### Thyroid scintigraphy

Thyroid scintigraphy allows the assessment of regional thyroid function and the detection of functioning nodules (95, 96). For routine use, most often 99mTc is used, based on a combination of low cost, wide availability, and low radiation burden. However, in a minority, this approach may lead to the misclassification of hypofunctioning as hyperfunctioning nodules (95). Thyroid scintigraphy should be performed when serum TSH is suppressed or at the lower normal limits. Of note, in areas of current or previous iodine deficiency, hyperfunctioning nodules may also be seen in individuals with normal TSH, meriting the use of thyroid scintigraphy (97, 98).

While we do not suggest [99mTc]Tc-MIBI imaging for routine use, it may be of value in case of indeterminate cytology, based on its relatively high negative predictive value for malignancy (99). Similarly, in patients with indeterminate cytology, 18F-fluoro-2-deoxy-D-glucose positron emission tomography ([^18^F]FDG-PET)/CT, although still debated, has shown promising results for excluding malignancy (100). Routine use of this resource, however, is limited in clinical practice by cost and limited accessibility.

Thyroid scintigraphy provides useful information in:

- solitary hyperfunctioning nodules, to avoid FNA biopsy, as hyperfunctioning nodules are rarely malignant;
- multinodular goiter, to differentiate hypofunctioning nodules suitable for FNA from hyperfunctioning lesions that do not need cytologic evaluation;
- to determine the eligibility for radioiodine therapy.

### Other imaging modalities

While there is no indication for initial evaluation of nodular thyroid disease using cross-sectional (i.e. contrast-enhanced CT and magnetic resonance imaging (MRI)) or functional (e.g. [^18^F]FDG-PET/CT) studies, incidentally detected thyroid lesions do have a ROM of 5–13% when using CT and MRI and of about 35% of high activity lesions when using [^18^F]FDG-PET/CT (33). These types of nodules should be investigated according to the diagnostic workup proposed in this guideline.

Neck and upper mediastinal CT scan should be performed in case of US or clinical suspicion of substernal extension. If using contrast media, the risk of thyrotoxicosis should be considered. The aim is to assess the extension into the upper mediastinum, the location and dimensions of the trachea, and the anatomical relation of the goiter to the oesophagus (34).

## Therapeutic options: non-surgical approaches

### Clinical surveillance

Most benign thyroid nodules are incidentally diagnosed and asymptomatic (4). In the absence of elevated TSH, the use of thyroid hormone in order to decrease TSH should be discouraged in order to limit the increased morbidity and mortality seen with such therapy (101, 102), but mainly because of its lack of efficacy in adequately decreasing size in symptomatic nodules (103). Iodine as well as selenium deficiency is associated with increased goiter prevalence. However, neither iodine (104) nor selenium supplementation (105) is recommended in iodine and selenium replete populations. During 1 year, significant growth, defined as an increase ≥20% in at least two nodule diameters with a minimum increase of 2 mm, or nodule volume increase >50%, occur only in a minority of cases (over a period of 5 years it is about 16%) (106). Thus, asymptomatic nodules should be followed based on the sonographic pattern and cytological assessment (Table 1, Fig. 2).

### Radioiodine therapy

An in-depth account is given in (107). Approximately 5–10% of solitary/dominant thyroid nodules are functioning on thyroid scintigraphy, with suppression of the peri-nodular thyroid tissue. Such nodules are, with extremely rare exceptions, benign, should not be biopsied, and are eligible for RAI treatment. Most patients are euthyroid or subclinically hyperthyroid at the time of diagnosis. Unless severely hyperthyroid or with cardiac comorbidity, individuals rarely need pre-treatment with anti-thyroid drugs. RAI is most often given as a fixed activity (e.g. 185–370 MBq), most often achieves euthyroidism, may cause hypothyroidism, and reduces nodule size by 30–50% in 12 months (108). Life-long follow-up is recommended.

The diagnostic workup of non-hyperfunctioning multinodular goiters, including FNA, should accord with the previously described algorithm (Figs. 1 and 2). When symptomatic and benign, thyroid nodules may, as an alternative to surgery, be eligible for RAI, especially in case of patients at surgical risk. Most patients are offered fixed activity RAI, dependent on local regulations. Hypothyroidism is rare (10–20% after 10 years), but life-long follow-up is recommended. Thyroid volume is typically reduced by 40% within 12 months and alleviates symptoms in most. In case of low RAI uptake and/or a large goiter, prestimulation with rhTSH has been demonstrated to augment thyroid volume reduction by 35% (109) and increases the smallest cross-sectional area of the trachea, improves pulmonary function, and reduces pressure symptoms (110).

### Minimally invasive techniques

MITs are out-patient procedures, performed under US guidance, for non-surgical management of thyroid lesions that cause local pressure symptoms or esthetic concerns (17, 18). MITs include ethanol ablation (EA), based on the direct injection of ethanol into a cystic cavity, and TA techniques, which use various energy sources: laser, radiofrequency, microwaves, or high-intensity focused ultrasound (17). MITs result in a relevant and long-lasting decrease of nodule volume (57–77% at 5 years) that is paralleled by improvement of local symptoms (111, 112) and disease-related quality of life (113). These treatments do not require general anesthesia, rarely cause complications, and even more rarely thyroid dysfunction (17). EA is preferred as an effective, safe, and inexpensive treatment for cystic (or predominantly cystic) symptomatic thyroid nodules (114) while TA procedures, due to their geometric and predictable volume of tissue destruction, are the first-line treatment for solid thyroid lesions (17). TA treatments should aim at a balance between nearly complete nodule destruction, to prevent late regrowth, and sparing of at-risk areas, to minimize the risk of complications. TA is an alternative to RAI and surgery in small hyperfunctioning nodules (<10 mL), while it performs poorer in larger nodules (115). In such cases, TA may be considered for patients who decline or are not candidates for RAI therapy or surgery. Currently, a major limitation of TA procedures use is their limited availability and lack of long-term data (10).

For MIT procedure indications, refer to recommendations (Table 1). For a more detailed account of the use of TA in benign nodules or even very low-risk thyroid carcinomas, we refer to the recent pertinent guidelines (17, 18, 116).

## Therapeutic options: surgical approach

This section is focused on providing the indications for the use of surgery in the management of thyroid nodules, rather than defining the extent of surgery and exploring the currently available surgical techniques and their advantages and disadvantages.

Currently, surgery is one of the possible management options in patients with nodular thyroid disease due to the development of sensitive diagnostic tools (US, FNA) enabling active surveillance and use of MIT (4). As detailed previously, any treatment decision, not only restricted to surgery, needs to take into consideration patient preference and available alternatives in a dialogue with the patient.

According to the management algorithm of the present guideline (Fig. 2), surgery may be appropriate in the following scenarios (117, 118, 119, 120):

- Symptomatic nodular thyroid disease, as an alternative option to MIT and RAI therapy.
- Nodules that have been classified as benign at cytology and/or low risk at US (i.e. EU-TIRADS 2 or 3) and become symptomatic over time.
- Nodules with indeterminate cytology (Bethesda class III and IV) that are not suitable for active surveillance (i.e. large size, high suspicion of malignancy on US, symptomatology).
- Nodules with a Bethesda class V and VI cytology.

For nodules of uncertain malignant potential (Bethesda class III–V cytology), surgery allows for a definitive diagnosis (117). Molecular test results (if available) should be considered prior to operation (121). For diseases limited to one lobe, lobectomy/hemithyroidectomy is recommended. If such a disease is diagnosed in a nodular goiter, near-total thyroidectomy should be considered. In benign lesions (Bethesda class II cytology), even if asymptomatic, surgical treatment may be considered for nodules ≥4 cm (due to the ROM and increased probability of a false negative FNA) (122, 123), symptomatic nodules (airway or esophagus compression), in case of cosmetic concern, and retro-clavicular and mediastinal extension. Surgery is one of the available therapeutic options, besides RAI and MIT, for hyperfunctioning nodules and toxic multinodular goiter (124).

## Diagnostic and therapeutic algorithm

Figures 1 and 2 and Table 1 summarize the diagnostic workup, the recommendations facing a newly diagnosed thyroid nodule and the following management strategies.

## Concluding remarks and future perspectives

The present document offers clinicians a guide to the rational management of nodular thyroid disease. Although very few nodules are malignant, the possibility of cancer represents the overriding concern when thyroid gland lesions are brought to our attention. Neck US is the central hub in the diagnostic algorithm aimed at stratifying the ROM, allowing guided FNA, and promoting the individualized management currently advocated. For the vast majority of patients, both tools provide a reliable foundation for defining the initial management strategy, whether follow-up, surgery or non-surgical treatment with MIT. However, in a number of cases, the estimates of the ROM will remain uncertain and the subsequent choices unsettled. In such cases, the clinicians’ experience and individualized choices based on patient-related factors and preferences represent the way forward. New tools are emerging in clinical practice, such as molecular testing, which are not yet generally available in Europe. While the analytic and clinical validity have been extensively studied, the ability of the molecular analysis to improve patient outcomes sufficiently to justify its incorporation into real-world clinical practice has yet to be proven. Such studies are warranted for years to come. The current focus is on developing an internationally endorsed lexicon of US findings allowing a common RSS (51), refining and implementing the rapidly evolving technology of non-surgical techniques, and involving disease-specific PRO tools, for example, the ThyPRO, in the management of our patients (125).

## Supplementary Materials

Supplementary Material

## Declaration of interest

Agnieszka Czarniecka, Gilles Russ, Fernando Schmitt, Paula Soares, and Tamas Solymosi have no conflicts of interest to disclose. Cosimo Durante has reported advisory board honoraria from EISAI, Eli Lilly, and Roche. Laszlo Hegedüs has reported consultancy fees from Berlin-Chemie, Horizon, IBSA, Lundbeck, Merck-Serono, and Novo Nordisk Foundation. Enrico Papini has reported advisory board honoraria from IBSA and Terumo and consultancy fees from Novo Nordisk. Ralf Paschke has reported ThyroSPEC™ license fees, received grant from Bayer, and advisory board honoraria from Bayer, Eisai, and Ipsen.

## Funding

This work did not receive any specific grant from any funding agency in the public, commercial, or not-for-profit sector.
