# Supplementary material for: 2023 European Thyroid Association Clinical Practice Guidelines for thyroid nodule management
Source: Eur Thyroid J. 2023 Aug 14;12(5):e230067. doi: 10.1530/ETJ-23-0067 (PMC10448590; doi:10.1530/ETJ-23-0067)
Supplement: Supplementary Material [file supplementary_material.pdf]

Appendix

ETA ultrasound lexicon[14]

Composition: proportion of soft tissue or fluid in a nodule

- ***Solid***: composed almost entirely of soft tissue with <10% of liquid
- ***Mixed predominantly solid***: liquid component ≥10% but <50% of the nodule volume
- ***Mixed predominantly cystic***: liquid component ≥50% but <90% of the nodule volume
- ***Cystic***: composed entirely or nearly entirely of liquid
- ***Spongiform appearance***: tiny cystic spaces separated by thin septa

Echogenicity of the nodule: always refers to solid parts of a nodule

Reference tissues

- echogenicity of the normal healthy thyroid differentiates iso/hyperechoic and hypoechoic nodules
- echogenicity of the muscle fibers with low adipose tissue content differentiates minimally/moderately and markedly hypoechoic nodules.

Types of nodules

- ***Mildly hypoechoic***: darker than the healthy thyroid, but less dark than the muscle fibers with low adipose tissue content of the surrounding strap muscles or sterno-cleido-thyroid muscle
- ***Iso/hyperechoic***: similar brightness/brighter than the healthy thyroid
- ***Markedly hypoechoic***: similarly dark or darker than the muscle fibers with low adipose tissue content of the surrounding strap muscles or sterno-cleido-thyroid muscle

Echotexture: characterizes the uniform or multiform appearance of the solid portion of a nodule

- ***Heterogeneous***
    - has both iso/hyperechoic and hypoechoic solid portions and the largest contiguous part of the minority component is significant, e.g. at least 5 mm
    - preferred naming: dominantly hypoechoic or dominantly iso/hyperechoic
    - preferred to handle according to the hypoechoic part
- ***Homogeneous***

Margin: refers to the outline of a single pathological nodule

- ***Smooth margin***: clear demarcation from the surrounding thyroid parenchyma
- ***Ill-defined margin***: lack of a clear demarcation from the surrounding thyroid parenchyma; ill-defined margins are distinct from irregular ones and do not alter significantly the nodule's risk category
- ***Irregular margin***:
    - presence of 1 or more sharp angles (spiculated) or 1 or more round protrusions (microlobulated) on the margin
    - the surface irregularity is not caused by other conditions (infiltration of thyroiditis or compression of a neighboring nodule or anatomy)
    - the distance between the most protruding part of the nodule and the rest of it must be at least 2 mm.

Shape (direction of growth): by defining the shape of a nodule, the largest diameters in the three axis should be compared (not the diameters in a selected section).

- Parallel orientation:

- the anteroposterior diameter of a nodule is less than (oval) or equal to (round) its transverse diameter on the transverse and longitudinal planes

- Nonparallel orientation:
  - the ratio of the anteroposterior-to-transverse diameter of a nodule is >1 (taller than wide) or the ratio of the anteroposterior-to-longitudinal diameter is >1 (taller than long). Some authors advocate the use of a 1.2 ratio as a cut-off value to increase specificity.
  - the nonparallel orientation cannot be explained by anatomical situation or the presence of the distorting effect of a neighbouring nodule

Intranodular hyperechoic figures

- ***Comet tail artifact***: echogenic foci showing comet-like echogenic tails generated by reverberation artifacts within the cystic component.
- ***Back-wall cystic figures***: bright echogenic lines and punctate granules in the dorsal wall of cystic areas caused by posterior enhancement
- ***Fibrosis***: coexistence of similarly bright echogenic granules and lines.
- ***Macrocalcification:*** defined by the presence of an acoustic shadow and caused by >1-mm coarse and large calcification which can be obscured.
  - ***Egg shell calcification***: a special form which is characterized by an echogenic line surrounding the nodule giving the appearance of a discrete calcified wall, along with marked posterior acoustic shadowing
- ***Microcalcification***: <1-mm, most often round, bright echogenic foci in the solid part of a nodule in the absence of similarly bright echogenic lines.

- *Special consideration*. We must be aware that the cystic content could be desiccated or can be very tiny, therefore, the distinction of a microcalcification from a comet-tail artifact or back wall figure is not always possible.

Extrathyroidal extension (ETE)

- Possible signs of ETE: discontinuation or bulging of the thyroid pseudocapsule and the degree of abutment does not exceed 50% of the nodule' perimeter
- ETE can be considered: if the thyroid pseudocapsule is discontinuous or not visible and the degree of abutment exceeds 50% of the nodule' perimeter
- ETE should be considered: if the thyroid pseudocapsule is discontinuous or not visible and the nodule bulges into the neighboring structures
- ETE can be reliably excluded in the absence of capsular contact

Vascularity:

- Type I: absence of intranodular or perinodular flow
- Type II: presence of perinodular and/or slight intranodular flow
- Type III: presence of marked intranodular flow
